# Photosensitizer and Charge Separator Roles of g-C₃N₄ Integrated into the CuO-Fe₂O₃ p-n Heterojunction Interface for Elevating PEC Water Splitting Potential

**DOI:** 10.3390/nano15070551

**Published:** 2025-04-04

**Authors:** Ramesh Reddy Nallapureddy, Sai Kumar Arla, Andrés Ibáñez, Durga Prasad Pabba, Jae Hak Jung, Sang Woo Joo

**Affiliations:** 1 School of Chemical Engineering, Yeungnam University, Gyeongsan 38541, Republic of Korea; rameshsun999@gmail.com; 2 Department of Physics, Yeungnam University, Gyeongsan 38541, Republic of Korea; saiarla853@gmail.com; 3 Departamento de Física, Facultad de Ciencias Físicas y Matemáticas, Universidad de Chile, Casilla 653, Santiago 8370451, Chile; aibanez@dfi.uchile.cl; 4 Departamento de Electricidad, Facultad de Ingeniería, Universidad Tecnológica Metropolitana (UTEM), Santiago 7800002, Chile; 5 School of Mechanical Engineering, Yeungnam University, Gyeongsan 38541, Republic of Korea

**Keywords:** CuO-Fe_2_O_3_@g-C_3_N_4_, ternary hybrid composite, micro-cubes, charge carriers, renewable energy

## Abstract

In sustainable hydrogen generation, photoelectrochemical (PEC) water splitting stands as a crucial technology, offering solutions to the global energy crisis while tackling environmental challenges. PEC water splitting relies on metal oxide nanostructures due to their unique electronic and optical characteristics. This research highlights the development of a CuO-Fe_2_O_3_@g-C_3_N_4_ nanocomposite, created through the integration of three components and fabricated via a one-pot hydrothermal process, precisely engineered to enhance PEC water-splitting efficiency. The combination of CuO, Fe_2_O_3_, and g-C_3_N_4_ results in a unified heterojunction structure that efficiently mitigates issues associated with charge carrier recombination and structural stability. Additionally, the analyses of both the structure and composition confirmed the precise synthesis of the composite. The CuO-Fe_2_O_3_@g-C_3_N_4_ nanocomposite achieved a photocurrent density of 1.33 mA cm^−2^ vs. Ag/AgCl upon exposure to light, demonstrating superior PEC performance and outperforming the individual CuO and Fe_2_O_3_ components. The enhanced performance is attributed to g-C_3_N_4_ acting as a photoactive material, generating charge carriers, while the combination of CuO-Fe_2_O_3_ enables efficient carrier separation and mobility. This synergistic interaction significantly enhances photocurrent generation and ensures long-term stability, positioning the material as a highly promising solution for sustainable hydrogen production. These results highlight the promise of hybrid nanocomposites in driving progress in renewable energy technologies, opening new avenues for the development of more efficient and long-lasting PEC systems.

## 1. Introduction

The quest for sustainable energy supply has become a vital objective, particularly in response to the escalating global energy demand and the rapid depletion of conventional energy reserves. Harnessing solar light for water splitting, resulting in hydrogen generation, emerges as a great potential solution to address this challenge [1,2]. As a plentiful and clean-natured fuel, hydrogen presents a promising option to replace fossil fuels, facilitating the transition to an additional maintainable and eco-conscious energy landscape [3,4,5]. Hydrogen is considered a key solution for the global transition to sustainable energy due to its potential as a clean, renewable fuel. It can be produced from a variety of sources, including water, and utilized in fuel cells to generate electricity without harmful emissions. As a versatile energy carrier, hydrogen plays a crucial role in decarbonizing industries, transportation, and power generation, contributing to the reduction of greenhouse gas emissions [6]. These solar-powered water-splitting systems are generally equipped with electrodes layered with semiconductors and photocatalytic materials and engineered with distinct characteristics for ensuring high-performance efficiency. The effectiveness of photonic absorption from sunlight, as well as charge carrier transport and separation within the system, is significantly enhanced by using materials with meticulously controlled morphologies and carefully tailored bandgap structures [7,8]. A key consideration in the development of such technologies is the demand for materials with well-defined morphologies. This approach involves the intentional engineering of materials at the nanoscale level to greatly expand the surface area exposed to sunlight for improved absorption. By increasing the availability of active sites for catalytic reactions, these refined morphologies substantially elevate system efficiency.

Metal oxide nanostructures (MONSs) have attracted considerable interest due to their promising applicability in PEC water-splitting technologies. [9,10,11,12]. Despite their promising attributes, these materials face critical challenges, including elevated recombination rates, insufficient light absorption, and durability challenges, all of which hinder their practical use in PEC systems. In order to tackle the identified obstacles, researchers focused on developing nanocomposites by integrating MONSs with complementary resources like carbon and LDHs [13,14]. These nanocomposites have shown improvements by lowering recombination rates and enhancing light absorption. However, stability remains a significant and unresolved challenge. Under PEC conditions, the degradation of these materials remains a major barrier to their effective application in water splitting. Addressing this issue requires sustained research and innovative solutions to improve the stability of these combined materials, opening new avenues for the development of more viable and high-performing PEC water-splitting systems.

To tackle stability issues, researchers are investigating advanced synthesis techniques, surface engineering, and innovative doping approaches. Therefore, establishing heterojunction interfaces between metal oxides has proven to be a powerful and promising approach for significantly improving PEC water-splitting performance [3,15]. These heterojunctions are vital in promoting charge separation and transfer, which helps to minimize charge carrier recombination that can negatively affect the effectiveness of PEC. Additionally, the interfaces tune the electronic band configuration of the MONSs, which is consequential in improved solar light capture over an extended wavelength spectrum [16,17].

In this, employing copper oxide (CuO) alongside iron (III) oxide (Fe_2_O_3_), both of which are recognized for their strong absorption in the visible-light spectrum, offers a compelling approach. Constructing a heterojunction interface between CuO and Fe_2_O_3_ emerges as a promising strategy for improving the performance [17,18]. CuO, owing to its lower bandgap and responsiveness to high-energy light, works in cooperation with Fe_2_O_3_, which is efficient in capturing photons from the visible-light region. This integrated photon absorption capability results in a synergistic interaction, effectively extending the range of solar wavelengths utilized. Moreover, the favorable alignment of energy states at the heterojunction interface enables effective dissociation and migration of charge carriers, thereby reducing recombination losses that typically hinder PEC efficiency. The integration of CuO, known for its cost-effectiveness and natural abundance, with Fe_2_O_3_, recognized for its robust absorption in the visible spectrum, renders the CuO-Fe_2_O_3_ heterojunction a highly suitable choice for large-scale PEC applications. This heterojunction not only enhances performance through synergistic effects but also improves stability by protecting against corrosion, making it highly promising for efficient PEC water splitting.

Incorporating graphitic carbon nitride (g-C_3_N_4_) into CuO-Fe_2_O_3_ heterojunctions holds great promise in PEC. As a two-dimensional polymer, g-C₃N₄ exhibits an appropriate energy bandgap tailored for capturing visible light, effectively complementing the light-harvesting capabilities of CuO and Fe_2_O_3_ [1,19]. It also acts as a supporting catalyst, enhancing charge carrier dissociation and suppressing recombination events, both of which are essential for boosting water-splitting performance. Additionally, g-C_3_N_4_ creates active catalytic regions at the junction between the heterostructured materials, promoting faster charge carrier transfer and improving the reaction kinetics [20]. Its excellent stability and tunable electronic properties ensure efficient band alignment with CuO and Fe_2_O_3_, facilitating optimal charge transfer [21]. Previous studies, such as the work of Pannan et al., have demonstrated the effectiveness of α-Fe_2_O_3_/CuO heterojunctions, reporting an achieved photocurrent output of 0.53 mA cm^−2^ measured at 0.1 V relative to the RHE [17]. Similarly, Dayu et al. fabricated a TiO_2_–RGO–CuO/Fe_2_O_3_ hybrid structure, which exhibited superior photocatalytic efficiency [22]. Jingyi et al. demonstrated that constructing a Fe_2_O_3_/CuO heterojunction photoanode enhanced the incident photon-to-current efficiency (IPCE) by a factor of 2.6 relative to unmodified Fe_2_O_3_ [23]. However, the integration of g-C_3_N_4_ with CuO/Fe_2_O_3_ remains largely unexplored, representing a new approach to further enhance PEC water-splitting efficiency.

In this study, we synthesized a CuO-Fe_2_O_3_/g-C_3_N_4_ heterojunction and evaluated its PEC water-splitting performance using LSV, chronoamperometry (I-t), and impedance spectroscopy. The Fe_2_O_3_/CuO heterojunction created an intrinsic electric field at the p-n junction, facilitating charge carrier migration and reducing electron–hole recombination, thus enhancing water-splitting efficiency. Additionally, the integration of g-C_3_N_4_ optimized charge separation, further improving the photocatalytic performance of the CuO-Fe_2_O_3_@g-C_3_N_4_ heterojunction, demonstrating its potential for PEC applications.

## 2. Experimental Work

### 2.1. Materials and Chemicals

Comprehensive information on the characterization methods employed in this study is provided in the Supporting Information section.

### 2.2. Synthesis of CuO-Fe_2_O_3_@g-C_3_N_4_ Ternary Composite

The CuO-Fe_2_O_3_@g-C_3_N_4_ three-component nanostructure was prepared via a single-step hydrothermal synthesis process. First, 5 mM of copper (II) acetate monohydrate (C_6_H_6_CuO_4_) were dissolved in 60 mL of deionized water, and the solution was stirred for a few minutes to ensure proper dissolution. After that, 8.45 mM of iron (III) chloride hexahydrate (FeCl_3_·6H_2_O) was gradually added, and the solution was stirred for another 10 min to allow for complete mixing of the metal precursors. This step is crucial to ensure a uniform distribution of both the copper and iron ions, which directly impacts the formation of the nanocomposite structure. Simultaneously, 0.25 g of g-C_3_N_4_ were sonicated in 5 mL of deionized water to ensure even dispersion of the material and added to the above solution. Following this, 0.7 g of hexamethylenetetramine (C_6_H_12_N_4_) were introduced into the mixture, acting as a stabilizing agent. The prepared solution was sealed in an autoclave and underwent hydrothermal processing at 150 °C for a duration of 8 h. This step facilitates the formation of the desired nanocomposite through controlled crystallization and particle growth. Once the reaction was complete, the resulting precipitate was carefully washed multiple times with deionized water and ethanol to eliminate any unreacted species or byproducts. The cleaned precipitate was then dried overnight at 80 °C in a hot-air oven to ensure the complete removal of moisture. In addition to the ternary composite, pristine CuO (without the iron precursor or g-C_3_N_4_), Fe_2_O_3_ (without the copper precursor or g-C_3_N_4_), and a binary CuO-Fe_2_O_3_ composite (without g-C_3_N_4_) were synthesized using the same procedure. This comparative synthesis allows for a better understanding of the influence of g-C_3_N_4_ and the heterojunction between CuO and Fe_2_O_3_ on the structural and functional properties of the final nanocomposites.

### 2.3. Characterization and PEC Water-Splitting Analysis

The details about the characterization techniques, electrode preparation, and PEC measurements are given in the Supporting Information.

## 3. Characterization Analysis

### 3.1. Crystallographic and Structural Features

**Figure 1:**
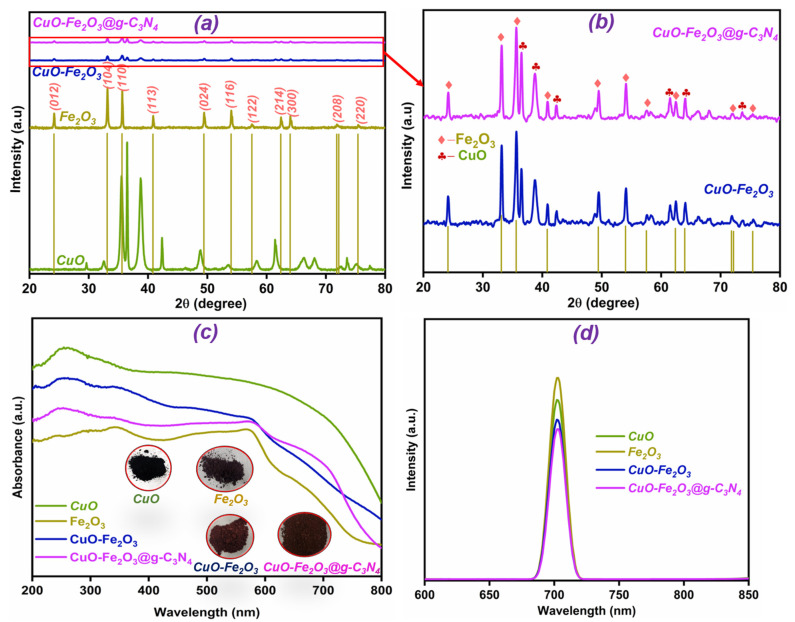
(**a**) XRD patterns of CuO, Fe_2_O_3_, CuO-Fe_2_O_3_, and CuO-Fe_2_O_3_@g-C_3_N_4_, (**b**) enlarged XRD view of the CuO-Fe_2_O_3_ and CuO-Fe_2_O_3_@g-C_3_N_4_ compounds, (**c**) UV-Vis diffuse reflectance spectra (DRS) with inset showing captured images of the fabricated materials, and (**d**) Fl spectra of CuO, Fe_2_O_3_, CuO-Fe_2_O_3_, and CuO-Fe_2_O_3_@g-C_3_N_4_.

X-ray diffraction (XRD) measurements were thoroughly performed to examine the crystallographic structure and phase composition of CuO, Fe_2_O_3_, CuO-Fe_2_O_3_, and CuO-Fe_2_O_3_@g-C_3_N_4_ samples. As shown in Figure 1a, the resulting 2θ peaks in CuO are consistent with the standard JCPDS reference pattern (Card No. 00-045-0937), validating the proper crystallization of CuO [24]. Additionally, peaks at 29.58°, 36.49°, and 42.38° suggest the presence of Cu_2_O [25]. Although these Cu_2_O peaks are present, CuO remains the dominant phase. The 2θ peaks align with the JCPDS (Card No. 00-024-0072) for Fe_2_O_3_, confirming its purity for Fe_2_O_3_ [26]. Figure 1a shows that the CuO-Fe_2_O_3_ and CuO-Fe_2_O_3_@g-C_3_N_4_ composites exhibit weakened and merged XRD peak intensities corresponding to those of the standalone CuO and Fe_2_O_3_ phases. A closer look at the XRD patterns in Figure 1b reveals distinct peaks for CuO and Fe_2_O_3_ within the composites, suggesting strong interfacial interactions between the two components. Upon introducing g-C_3_N_4_ into the CuO-Fe_2_O_3_ composite, the peak intensities decrease further, accompanied by slight broadening and peak shifts. These modifications could be attributed to shifts in the crystallite dimensions or lattice strain introduced by the integration of g-C₃N₄ [27]. The absence of identifiable g-C_3_N_4_ peaks could be due to its lower concentration or weaker XRD signals within the composite.

### 3.2. Optical Characteristics

The light-responsive behavior of the fabricated materials was analyzed to assess their potential applicability in PEC water-splitting processes. The UV-Vis DRS spectra were recorded for the synthesized materials. As shown in Figure 1c, CuO exhibited an absorption edge in the visible spectrum, aligning with its known capability to harness solar energy for photocatalytic applications, making it a suitable candidate for PEC processes [28,29]. Also, Fe_2_O_3_ exhibited a clear absorption edge within the visible light range, with its starting point and peak position reflecting its anticipated efficiency in photocatalytic applications and its capability to absorb visible light effectively [30]. A significant redshift in the absorption edge was detected for the CuO-Fe_2_O_3_ composite compared to the individual materials. This shift indicates strong interactions between CuO and Fe_2_O_3_, which modify the composite’s electronic structure and improve its light absorption properties. The broader absorption range of the composite highlights its improved potential for photocatalytic activity associated with the standalone resources. Additionally, the addition of g-C_3_N_4_ into the CuO-Fe_2_O_3_ composite expanded the absorption spectrum even further into the visible region, indicating an improvement in its ability to capture more of the solar spectrum. The shift in the absorption edge toward longer wavelengths compared to the CuO-Fe_2_O_3_ composite points to additional modifications in the electronic structure due to the presence of @g-C_3_N_4_. The inclusion of g-C_3_N_4_ not only enhances light absorption but also suggests better charge separation, further contributing to the composite’s enhanced photocatalytic performance, particularly for PEC water-splitting applications [31,32]. Digital photographs of the samples are shown in the inset of Figure 1c.

Understanding charge carrier recombination rates is vital for evaluating the PEC efficiency of synthesized materials. To investigate these rates, fluorescence (FL) spectroscopy was employed, as depicted in Figure 1d. The FL intensity for CuO was relatively high, indicating a significant level of charge carrier recombination. Fe_2_O_3_ also showed distinct FL characteristics, reflecting its recombination behavior and the associated charge transfer limitations. Notably, the FL intensities of the CuO-Fe_2_O_3_ and CuO-Fe_2_O_3_@g-C_3_N_4_ composites were markedly lower than those of the pure CuO and Fe_2_O_3_ samples. This decrease in FL intensity suggests a reduction in charge carrier recombination, likely due to the formation of a heterojunction between CuO and Fe_2_O_3_. The interface between these two materials enhances charge separation and transfer efficiency, reducing recombination rates in contrast to the unmodified counterparts. Moreover, the primer of g-C_3_N_4_ to the CuO-Fe_2_O_3_ compound further influenced the FL spectra, reflecting alterations in the recombination of photogenerated charge carriers. The presence of g-C_3_N_4_ facilitates a more effective charge separation by providing additional pathways for electron transfer, thereby enhancing the overall charge transport properties of the system. The observed reduction in FL intensity and the spectral modifications induced by g-C_3_N_4_ imply improved charge separation and a significant decrease in recombination, which are key factors contributing to the enhanced photocatalytic performance of the CuO-Fe_2_O_3_@g-C_3_N_4_ ternary nanocomposite. This improvement in charge dynamics underscores the potential of the composite for efficient PEC water splitting and other solar-driven applications [33,34].

### 3.3. Morphological Properties

**Figure 2:**
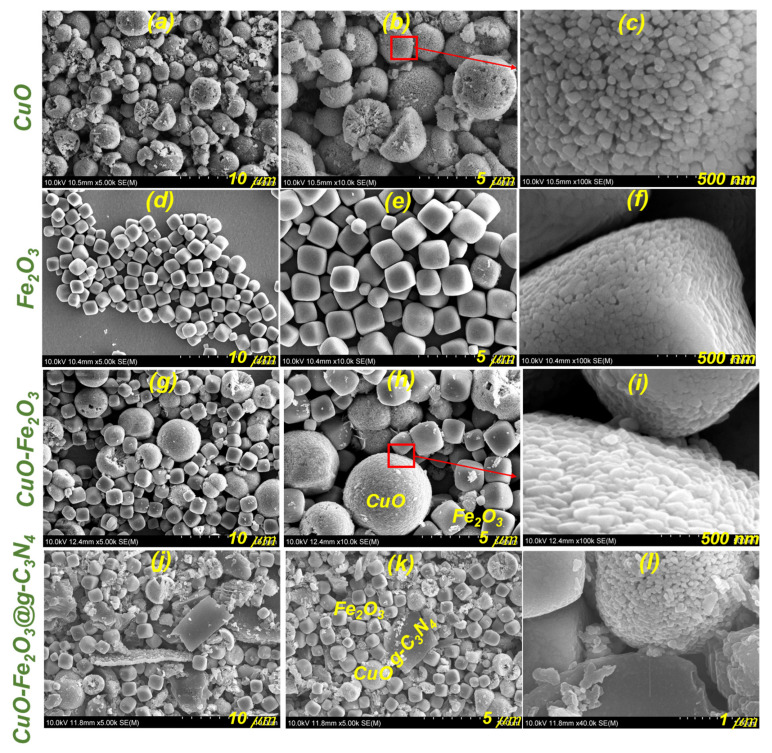
FE-SEM micrographs showing (**a**–**c**) spherical CuO, (**d**–**f**) Fe_2_O_3_ cubes, (**g**–**i**) CuO-Fe_2_O_3_ binary composite, and (**j**–**l**) ternary composite.

Figure 2 presents key morphological details of the synthesized materials as revealed by field emission scanning electron microscopy (FE-SEM) analysis. The CuO images shown in Figure 2a–c illustrate the development of microspheres formed by the accumulation of smaller nanospheres, likely driven by self-organizing mechanisms affected by intermolecular forces. The structured microspheres offer benefits like a larger surface area and distinct optical properties, making them well-suited for applications such as PEC water splitting. The FE-SEM images for Fe_2_O_3_ shown in Figure 2d–f display microcubes formed by the accumulation of smaller nanoparticles. This formation implies a sophisticated interplay of nucleation and growth, where the microcubes show an organized self-assembly with particular crystallographic patterns. These structural traits boost the material’s potential in photocatalytic applications. The morphology of the CuO-Fe_2_O_3_ composite (Figure 2g–i) shows a combination of features from both materials, demonstrating effective integration. The contact between CuO and Fe_2_O_3_ at the interface is crucial, as it fosters synergistic effects that improve the composites in the recombination of their photogenerated charge carrier behavior. This robust interface coupling plays a vital role in facilitating effective charge migration and dissociation, both of which are essential for improving the photocatalytic performance of the composite material. These improved charge dynamics significantly increase the material’s overall effectiveness in processes for applications like PEC water decomposition and other catalytic applications. The CuO-Fe_2_O_3_@g-C_3_N_4_ composite (Figure 2j–l) exhibits a noticeable sheet-like structure of g-C_3_N_4_, indicating a strong interaction with CuO and Fe_2_O_3_. This structure suggests that g-C_3_N_4_ is well-dispersed within the composite, promoting effective charge transfer and separation, which are critical for improving the utilization of solar energy for photocatalytic transformation processes. Moreover, g-C_3_N_4_’s presence enhances the material’s ability to absorb light and prolongs charge carrier lifetimes, further boosting the overall efficiency of the photocatalytic process. The EDX elemental mapping of the CuO-Fe_2_O_3_@g-C_3_N_4_ nanocomposite (Figure S1) provides critical insights into the distribution and homogeneity of key elements within the composite structure. The mapping images in Figure S1b–f clearly demonstrate the uniform distribution of copper (Cu), iron (Fe), oxygen (O), carbon (C), and nitrogen (N) elements across the surface of the composite, confirming the successful integration of all components. This homogeneity is essential for ensuring the effectiveness of the composite in photocatalytic applications, where the uniform presence of each element plays a key role in optimizing the material’s overall performance.

**Figure 3:**
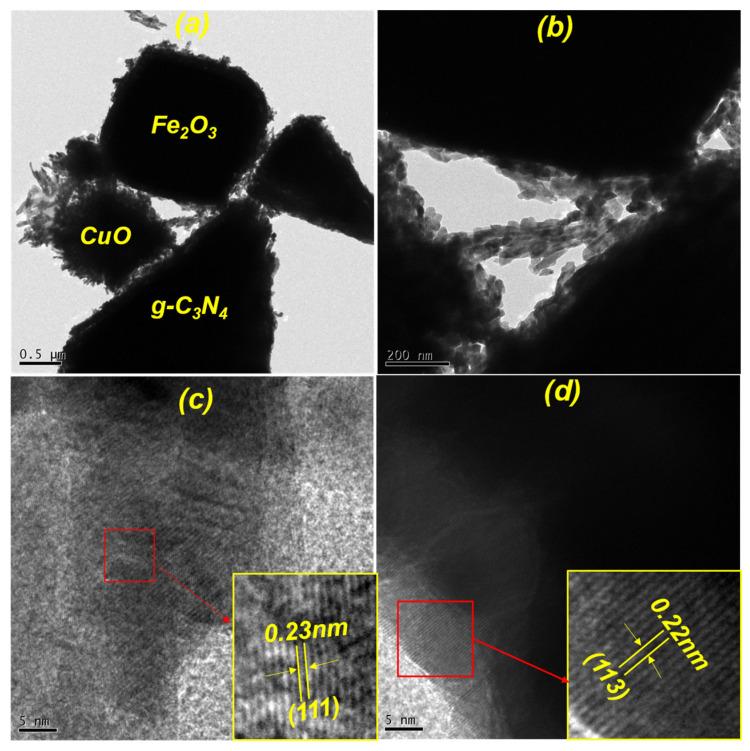
(**a**,**b**) TEM micrographs of the CuO-Fe_2_O_3_@g-C_3_N_4_ ternary nanocomposite; (**c**,**d**) HR-TEM images featuring inset IFFT patterns of CuO and Fe_2_O_3_, indicating their corresponding interplanar spacing values.

To examine the surface structure and crystalline nature of the CuO-Fe_2_O_3_@g-C_3_N_4_ compound, transmission electron microscopy (TEM) was conducted. This technique provided more detailed insights into the material’s structure, complementing the findings obtained from the FE-SEM analysis. As shown in Figure 3a, the spherical shape of CuO, the cubic structure of Fe_2_O_3_, and the sheet-like formation of g-C_3_N_4_ are clearly visible. The morphological characteristics identified through TEM are in agreement with those observed in the FE-SEM analysis, ensuring consistency across both methods. As depicted in Figure 3b, the TEM image illustrates the robust interface between CuO, Fe_2_O_3_, and g-C_3_N_4_, which plays a significant role in boosting PEC water-splitting efficiency by enabling efficient charge separation and transfer. The morphological characteristics identified through TEM are in agreement with those observed in the FE-SEM analysis, ensuring consistency across both methods. As depicted in Figure 3b, the TEM image illustrates the robust interface between CuO, Fe_2_O_3_, and g-C_3_N_4_, which plays a significant role in boosting the PEC water-splitting efficiency by enabling efficient charge separation and transfer. In addition, HR-TEM images, illustrated in Figure 3c,d, highlight the crystallinity of CuO and Fe_2_O_3_ by revealing their distinct lattice fringes. The inverse fast Fourier transform (IFFT) images, shown as insets in these figures, depict lattice spacings of 0.23 nm and 0.22 nm, which are attributed to the (111) plane of CuO and the (113) plane of Fe_2_O_3_, respectively [35,36]. These findings confirm the superior crystallinity of the fabricated materials by providing precise insights into their lattice arrangements and crystallographic orientations. The distinct interfacial contact and well-ordered crystal structure highlight the effectiveness of the mixture and design of the composite, which is crucial for improving its PEC water-splitting performance.

### 3.4. X-Ray Photoelectron Spectroscopy (XPS) Analysis

**Figure 4:**
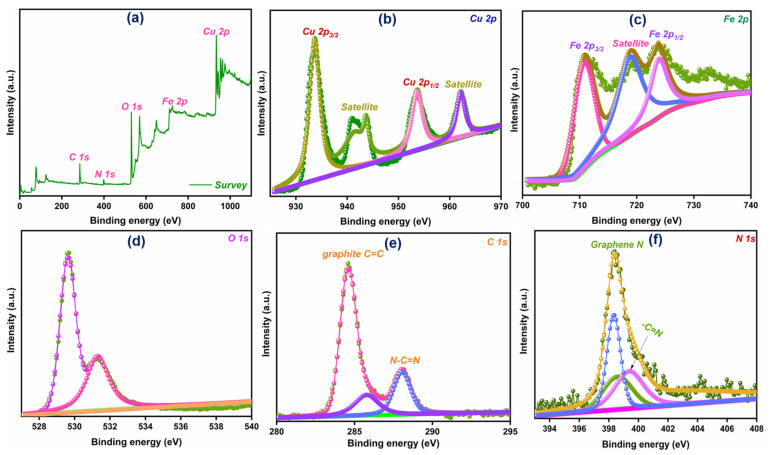
XPS analysis of the CuO-Fe_2_O_3_@g-C_3_N_4_ ternary system, including (**a**) the complete survey scan and high-resolution fitted spectra of (**b**) Cu 2p, (**c**) Fe 2p, (**d**) O 1s, (**e**) C 1s, and (**f**) N 1s regions.

XPS was employed to analyze the oxidation states and elemental composition of the CuO-Fe_2_O_3_@g-C_3_N_4_ compound. The XPS survey spectrum depicted in Figure 4a verifies the existence of essential elements such as Cu, Fe, O, C, and N. The Cu 2p XPS (Figure 4b) displays characteristic peaks at 932.2 eV and 952.1 eV, which are attributed to Cu 2p_3/2_ and Cu 2p_1/2_ transitions. In addition, the appearance of satellite features at 942.5 eV and 961.3 eV provides further evidence of the CuO phase existing on the material’s surface [37,38]. The Fe 2p_3/2_ and Fe 2p_1/2_ peaks are displayed at 710.9 eV and 723.8 eV, as shown in Figure 4c, which confirms the oxidation state of iron as Fe(III) [39]. The O 1s XPS spectrum (Figure 4d) shows a peak at 529.61 eV corresponding to lattice oxygen, whereas the signal at 531.6 eV is associated with surface-adsorbed oxygen, typically present in hydroxyl functionalities [40]. The data offer a comprehensive insight into the various forms of oxygen incorporated in the catalyst structure, contributing to its overall functionality [41,42]. In the C 1s spectrum (Figure 4e), the peak is attributed to C=C bonds arising from adventitious surface carbon, and sp^2^-hybridized carbon in the N–C=N groups [43]. The N 1s spectrum (Figure 4f) shows two deconvoluted peaks at 398.38 eV and 399.4 eV, corresponding to pyridinic and pyrrolic nitrogen, respectively. These signals underscore the variety of nitrogen configurations within the composite, suggesting the existence of several distinct nitrogen-based functional groups [44,45]. The deconvoluted XPS spectra of Fe 2p and Cu 2p are shown in Figure S2 in the Supporting Information. The XPS spectra reveal noticeable differences between the individual components and the composite, particularly in the binding energy shifts and peak intensities. These differences are indicative of chemical interactions at the interfaces of CuO, Fe_2_O_3_, and g-C_3_N_4_. For instance, binding energy shifts observed in the Fe 2p and Cu 2p peaks suggest altered electronic environments in the composite compared to the individual phases. These shifts are commonly attributed to electron transfer or charge redistribution at the heterojunction interface, which can occur when the components interact [46].

## 4. PEC Water-Splitting Analysis

**Figure 5:**
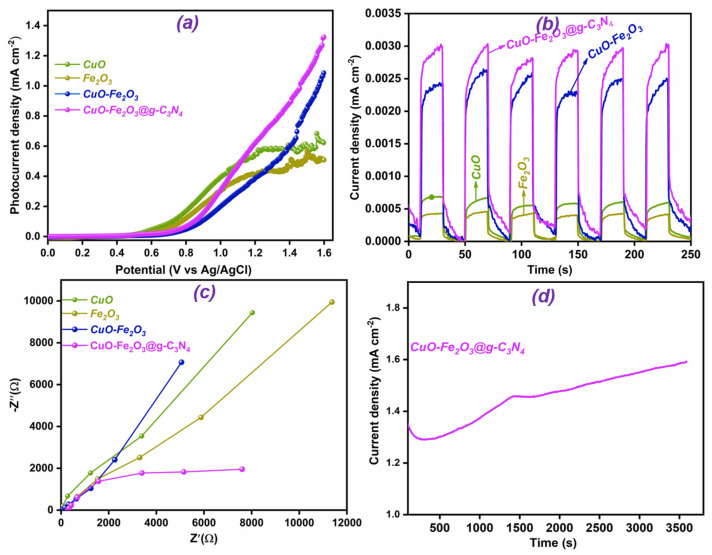
(**a**) Linear sweep voltammetry (LSV) results, (**b**) chronoamperometry (i–t) curves under intermittent light conditions, and (**c**) Nyquist impedance plots for CuO, Fe_2_O_3_, CuO-Fe_2_O_3_, and CuO-Fe_2_O_3_@g-C_3_N_4_, (**d**) stability performance of CuO-Fe_2_O_3_@g-C_3_N_4_ under continuous light exposure for up to 1 h.

A photoelectrochemical (PEC) water-splitting evaluation, involving linear sweep voltammetry (LSV), current–time (i–t) measurements, and electrochemical impedance spectroscopy (EIS), was performed on fabricated photoelectrodes. The LSV is crucial for assessing the photocurrent response in PEC, providing insight into the efficiency of photoelectrode materials. Therefore, LSV was performed on CuO, Fe_2_O_3_, CuO-Fe_2_O_3_, and CuO-Fe_2_O_3_@g-C_3_N_4_ photoelectrodes under illumination, and the results are depicted in Figure 5a. The resultant photocurrent densities, such as 0.62, 0.5, 1.09, and 1.33 mA cm^−2^, correspond to the CuO, Fe_2_O_3_, CuO-Fe_2_O_3_, and CuO-Fe_2_O_3_@g-C_3_N_4_ photoelectrodes, respectively. Among all photoelectrodes, the CuO-Fe_2_O_3_@g-C_3_N_4_ photoelectrodes showed significantly better performance than the individual materials and moderately higher performance than the CuO-Fe₂O₃ composite electrode. The remarkable performance of the CuO-Fe_2_O_3_@g-C_3_N_4_ ternary composite in PEC water splitting can be attributed to multiple advanced mechanisms. A primary driver is the establishment of a type-II band alignment heterostructure between CuO and Fe_2_O_3_, which significantly enhances the charge separation and accelerates the charge transfer processes [47,48]. This efficient separation is critical for minimizing electron–hole recombination, thereby amplifying photocurrent generation and ensuring that a greater number of charge carriers contribute to the water-splitting reaction. Additionally, g-C_3_N_4_ plays a pivotal role in improving the light absorption capacity of the composite, particularly within the visible-light spectrum [20,49]. Its ability to absorb a wider range of photons leads to a higher generation rate of electron–hole pairs, thus boosting the overall efficiency of the PEC system [50]. The light-harvesting efficiency of g-C_3_N_4_ synergizes with the charge transport properties of CuO and Fe_2_O_3_, optimizing the photoelectrode’s overall PEC performance. Moreover, the cooperative interaction among CuO, Fe_2_O_3_, and g-C_3_N_4_ not only enhances the efficiency of the charge dynamics but also improves the structural stability and resilience of the composite [51,52].

The synthesized photoelectrodes, i.e., CuO, Fe_2_O_3_, CuO-Fe_2_O_3_, and CuO-Fe_2_O_3_@g-C_3_N_4_, were evaluated using chronoamperometry (i–t) under intermittent light, with 25 s light and dark cycles over a total time span of 250 s. This method was employed to evaluate the transient PEC response of the electrodes under water-splitting conditions. Periodic illumination provided real-time understanding of the charge dynamics associated with each photoelectrode. The experimental changes in current were associated with the charge accumulation and release processes at the electrode–electrolyte interface, influenced by the light on and off cycles. These alternating light conditions enabled continuous tracking of the system’s behavior in reaction to illumination changes, providing a deeper understanding of the processes governing charge accumulation and discharge inside the materials [53,54]. As depicted in Figure 5b, all the photoelectrodes demonstrated a strong response to light, showing rapid increases and decreases in current during the on and off cycles. The observed photocurrent density patterns were in agreement with the results obtained from LSV, further confirming the superior performance of the CuO-Fe_2_O_3_@g-C_3_N_4_ composite. Over the course of the 250 s experiment, the electrodes consistently displayed stable photocurrent densities with minimal signs of degradation, highlighting their strong stability. This consistent performance is ascribed to the inherent material properties combined with cooperative interactions occurring at the heterojunction interfaces [55,56]. A detailed comparison of photocurrent densities for the CuO, Fe_2_O_3_, and g-C_3_N_4_-based electrodes is provided in Table S1, further highlighting the superior stability and efficiency of the CuO-Fe_2_O_3_@g-C_3_N_4_ composite.

The electrochemical impedance spectroscopy (EIS) results offer crucial understanding of the charge transport dynamics and recombination pathways within the fabricated materials, as shown in Figure 5c. The unmodified CuO and Fe_2_O_3_ samples show greater charge transfer resistance than the CuO-Fe_2_O_3_ and CuO-Fe_2_O_3_@g-C_3_N_4_ composites. This elevated resistance in the pristine materials is primarily due to the limited availability of charge conduction pathways in single-phase structures, whereas the combination of materials in composites improves conductivity. Additionally, the low electrical conductivity of CuO and Fe_2_O_3_ is due to their specific band structures, which restrict charge carrier movement and hinder efficient charge transport [57,58]. In contrast, resulting from the formation of a heterostructure within the CuO-Fe_2_O_3_, this makes advantageous interactions that improve charge transport efficiency by providing smoother electron pathways. The interaction between CuO and Fe_2_O_3_ optimizes the material’s electronic configuration, thereby improving the overall electrical conductivity. The construction of a heterostructured interface linking CuO and Fe_2_O_3_ enhances charge separation and promotes more efficient electron transfer pathways, which is a key feature in many composite photoelectrodes used in PEC applications [59]. Furthermore, the incorporation of g-C_3_N_4_ in the CuO-Fe_2_O_3_@g-C_3_N_4_ composite further reduces charge transfer resistance, as the nitrogen present in g-C_3_N_4_ can enhance the conductivity by altering charge carrier concentrations and introducing additional pathways for charge transport. Nitrogen functionalities are known to improve the electronic properties of materials by modifying the density of states and facilitating the movement of charge carriers [60].

The stability of the CuO-Fe_2_O_3_@g-C_3_N_4_ composite was carefully tested under one hour of continuous light irradiation, as illustrated in Figure 5d, showing outstanding stability throughout the experiment. The observed behavior in Figure 5d, where the current density initially decreased and then increased, can be attributed to the activation process of the photocatalytic system. During the initial phase, the system may experience surface charge accumulation or electrochemical stabilization as it adjusts to the light exposure. This initial decrease in current density is often observed as the system stabilizes and the charge carriers reach a more stable state. As the system reaches its optimal operating conditions, the current density increases, reflecting the improved photocatalytic efficiency and more efficient charge transfer. Such transient behavior is commonly seen in photocatalytic systems and indicates the material’s ability to stabilize and enhance its performance over time [61]. For instance, Yun et al. (2025) [62] observed a similar behavior in their stability tests, where the current density initially decreased during the early stage of the test, followed by a gradual increase, which was attributed to the activation process of the photocatalytic system. They noted that the decreased activity was due to surface chemical variation [62]. Later, the current density gradually increases. This impressive performance is largely due to the synergistic interaction of CuO, Fe_2_O_3_, and g-C_3_N_4_ within the composite structure, which not only enhances the charge transfer efficiency but also reinforces the material’s structural integrity and durability [12,63].

**Figure 6:**
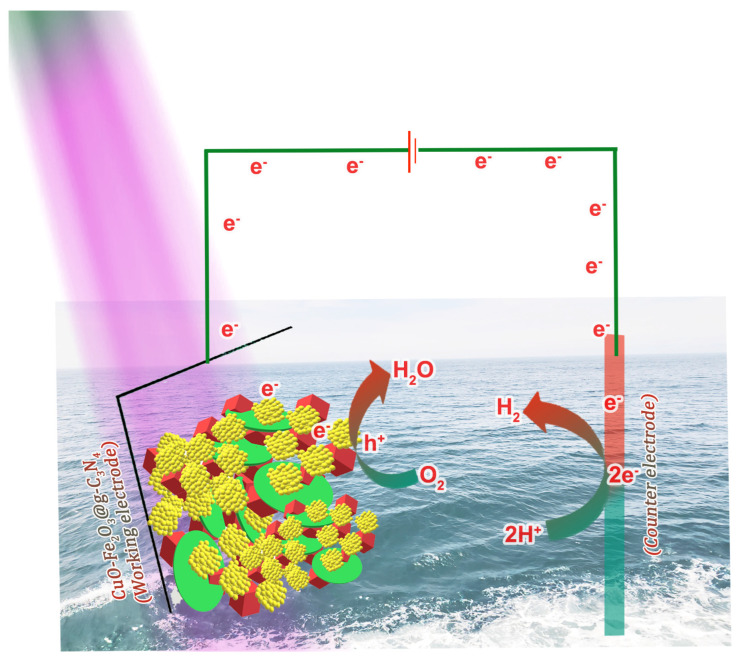
Possible PEC water-splitting mechanism using the CuO-Fe_2_O_3_@g-C_3_N_4_ electrode.

The bandgap characteristics of CuO and Fe_2_O_3_ reveal that the CuO conduction band edge sits considerably higher than that of Fe_2_O_3_. With the CuO functioning as a p-type semiconductor and the Fe_2_O_3_ as an n-type semiconductor, their combination results in the formation of a type-II heterojunction (Figure 6). This configuration aligns with Anderson’s model for p-n heterojunctions and promotes effective charge separation. This configuration proves highly beneficial for PEC applications, as it enhances charge carrier mobility while minimizing recombination. The improved charge separation resulting from the type-II heterojunction significantly boosts the overall efficiency, making it an effective system for PEC processes. In this composite, g-C_3_N_4_ acts as a key photosensitizer, efficiently capturing light energy to produce electron–hole pairs upon light exposure. When the composite is illuminated, g-C_3_N_4_ captures light, creating excited electrons in the conduction band and holes in the valence band. The type-II heterojunction between CuO and Fe_2_O_3_ promotes the directional flow of these charge carriers. This movement of electrons and holes enables efficient water-splitting reactions. The electrons in CuO are then used to reduce water, generating hydrogen ions, while the holes present in Fe_2_O_3_ participate in the oxidation of water, producing oxygen. The addition of g-C_3_N_4_ significantly improves the system’s overall PEC performance by not only increasing the absorption of light across a wider range of the spectrum but also enhancing the separation and transfer of photo-generated charges. The combined effect of the heterojunction between CuO and Fe_2_O_3_, along with g-C_3_N_4’_s light-harvesting properties, results in a highly efficient system for PEC water splitting. The composite allows for improved charge mobility and reduces recombination losses, making it an effective design for sustainable hydrogen production.

## 5. Conclusions

In this study, the CuO-Fe_2_O_3_@g-C_3_N_4_ photoelectrode was successfully created using a one-pot hydrothermal method. The photoelectrode showed a photocurrent density of 1.33 mA cm^−2^ (vs. Ag/AgCl) at 1.6 V under light exposure. This impressive performance is attributed to the synergistic interaction at the CuO-Fe_2_O_3_ heterojunction, which helps in efficient charge transfer, reducing the loss of electrons and holes. In addition to that, the g-C_3_N_4_ plays a key role in capturing light, boosting the amount of energy that can be used for water splitting. The different shapes of the nanostructures, such as CuO spheres, Fe_2_O_3_ cubes, and g-C_3_N_4_ sheets, provide more surface area for the reaction, improving the overall efficiency. The unique morphologies of the individual components, such as the CuO spheres, Fe_2_O_3_ cubes, and the g-C_3_N_4_ sheets, provide a high density of active sites that are essential for boosting the surface reactions during water splitting. The CuO spheres offer strong redox activity. Meanwhile, Fe_2_O_3_ provides additional photoactivity under visible light, and g-C_3_N_4_ facilitates charge separation and transport. The combination of these materials results in a composite that not only exhibits high photocurrent density but also demonstrates remarkable stability over long-term operation under continuous light exposure. This stability is critical for practical applications, as it ensures the composite can function effectively over extended periods without significant degradation. This combination of CuO, Fe_2_O_3_, and g-C_3_N_4_ into a single material marks an important step forward in the design of photoelectrodes for water splitting. In future studies, researchers should focus on producing this composite in larger quantities and testing it under different conditions, such as varying light intensity or electrolyte types. Additionally, exploring ways to further increase its efficiency and stability, such as by adding other elements or protective layers, could lead to even better results for sustainable hydrogen production through PEC water splitting.

## Data Availability

The original contributions presented in this study are included in the article/Supplementary Materials. Further inquiries can be directed to the corresponding authors.

## Supplementary Materials

The following supporting information can be downloaded at: https://www.mdpi.com/article/10.3390/nano15070551/s1. References [64,65,66,67,68,69,70,71,72] are cited in the Supplementary Materials.

## References

[1] Mohamed N.A., Safaei J., Ismail A.F., Khalid M.N., Mohd Jailani M.F.A., Noh M.F.M., Arzaee N.A., Zhou D., Sagu J.S., Teridi M.A.M. (2020). Boosting Photocatalytic Activities of BiVO_4_ by Creation of g-C_3_N_4_/ZnO@BiVO_4_ Heterojunction. Mater. Res. Bull..

[2] Jiao Z., Guan X., Wang M., Wang Q., Xu B., Bi Y., Zhao X.S. (2019). Undamaged Depositing Large-Area ZnO Quantum Dots/RGO Films on Photoelectrodes for the Construction of Pure Z-Scheme. Chem. Eng. J..

[3] Yadav J., Singh J.P. (2022). WO_3_/Ag_2_S Type-II Hierarchical Heterojunction for Improved Charge Carrier Separation and Photoelectrochemical Water Splitting Performance. J. Alloys Compd..

[4] Sharma P., Jang J.W., Lee J.S. (2019). Key Strategies to Advance the Photoelectrochemical Water Splitting Performance of α-Fe_2_O_3_ Photoanode. ChemCatChem.

[5] Liao J., Feng Y., Zhang X., Huang L., Huang S., Liu M., Liu Q., Li H. (2021). CuO-Co_3_O_4_ Composite Nanoplatelets for Hydrolyzing Ammonia Borane. ACS Appl. Nano Mater..

[6] Yu J., Li Z., Liu T., Zhao S., Guan D., Chen D., Shao Z., Ni M. (2023). Morphology Control and Electronic Tailoring of CoxAy (A = P, S, Se) Electrocatalysts for Water Splitting. Chem. Eng. J..

[7] Guo B.Y., Zhang X.Y., Ma X., Chen T.S., Chen Y., Wen M.L., Qin J.F., Nan J., Chai Y.M., Dong B. (2020). RuO_2_/Co_3_O_4_ Nanocubes Based on Ru Ions Impregnation into Prussian Blue Precursor for Oxygen Evolution. Int. J. Hydrogen Energy.

[8] Zarezadeh S., Habibi-Yangjeh A., Mousavi M., Ghosh S. (2020). Synthesis of Novel P-n-p BiOBr/ZnO/BiOI Heterostructures and Their Efficient Photocatalytic Performances in Removals of Dye Pollutants under Visible Light. J. Photochem. Photobiol. A Chem..

[9] Manh Hung N., Thi Bich V., Duc Quang N., Tien Hiep N., Nguyen C.V., Majumder S., Tien Hung P., Dinh Hoat P., Van Hoang N., Minh Hieu N. (2023). CuS–CdS@TiO_2_ Multi-Heterostructure-Based Photoelectrode for Highly Efficient Photoelectrochemical Water Splitting. Ceram. Int..

[10] Reddy N.R., Reddy P.M., Jyothi N., Kumar A.S., Jung J.H., Joo S.W. (2023). Versatile TiO_2_ Bandgap Modification with Metal, Non-Metal, Noble Metal, Carbon Material, and Semiconductor for the Photoelectrochemical Water Splitting and Photocatalytic Dye Degradation Performance. J. Alloys Compd..

[11] Hernández S., Cauda V., Chiodoni A., Dallorto S., Sacco A., Hidalgo D., Celasco E., Pirri C.F. (2014). Optimization of 1D ZnO@TiO_2_ Core-Shell Nanostructures for Enhanced Photoelectrochemical Water Splitting under Solar Light Illumination. ACS Appl. Mater. Interfaces.

[12] Zhang J., Ma H., Liu Z. (2017). Highly Efficient Photocatalyst Based on All Oxides WO_3_/Cu_2_O Heterojunction for Photoelectrochemical Water Splitting. Appl. Catal. B Environ..

[13] Ning F., Shao M., Xu S., Fu Y., Zhang R., Wei M., Evans D.G., Duan X. (2016). TiO_2_/Graphene/NiFe-Layered Double Hydroxide Nanorod Array Photoanodes for Efficient Photoelectrochemical Water Splitting. Energy Environ. Sci..

[14] Zhang Z., Sun L., Wu Z., Liu Y., Li S. (2020). Facile Hydrothermal Synthesis of CuO-Cu_2_O/GO Nanocomposites for the Photocatalytic Degradation of Organic Dye and Tetracycline Pollutants. New J. Chem..

[15] Fang G., Liu Z., Han C. (2020). Enhancing the PEC Water Splitting Performance of BiVO_4_ Co-Modifying with NiFeOOH and Co-Pi Double Layer Cocatalysts. Appl. Surf. Sci..

[16] Zhang J., Zhu Q., Wang L., Nasir M., Cho S.H., Zhang J. (2020). g-C_3_N_4_/CoAl-LDH 2D/2D Hybrid Heterojunction for Boosting Photocatalytic Hydrogen Evolution. Int. J. Hydrogen Energy.

[17] Kyesmen P.I., Nombona N., Diale M. (2021). Heterojunction of Nanostructured α-Fe_2_O_3_/CuO for Enhancement of Photoelectrochemical Water Splitting. J. Alloys Compd..

[18] Xu Q., Zhang Z., Song X., Yuan S., Qiu Z., Xu H., Cao B. (2017). Improving the Triethylamine Sensing Performance Based on Debye Length: A Case Study on A-Fe_2_O_3_@NiO(CuO) Core-Shell Nanorods Sensor Working at near Room-Temperature. Sens. Actuators B Chem..

[19] Murugan C., Karnan M., Sathish M., Pandikumar A. (2020). Construction of Heterostructure Based on Hierarchical Bi_2_MoO_6_ and g-C_3_N_4_ with Ease for Impressive Performance in Photoelectrocatalytic Water Splitting and Supercapacitor. Catal. Sci. Technol..

[20] Wen P., Sun Y., Li H., Liang Z., Wu H., Zhang J., Zeng H., Geyer S.M., Jiang L. (2020). A Highly Active Three-Dimensional Z-Scheme ZnO/Au/g-C_3_N_4_ Photocathode for Efficient Photoelectrochemical Water Splitting. Appl. Catal. B Environ..

[21] Ghane N., Sadrnezhaad S.K., Hosseini H. S.M. (2020). Combustion Synthesis of g-C_3_N_4_/Fe_2_O_3_ Nanocomposite for Superior Photoelectrochemical Catalytic Performance. Appl. Surf. Sci..

[22] Li D., Liang Z., Zhang W., Dai S., Zhang C. (2021). Preparation and Photocatalytic Performance of TiO_2_-RGO-CuO/Fe_2_O_3_ Ternary Composite Photocatalyst by Solvothermal Method. Mater. Res. Express.

[23] Ma J., Wang Q., Li L., Zong X., Sun H., Tao R., Fan X. (2021). Fe_2_O_3_ Nanorods/CuO Nanoparticles p-n Heterojunction Photoanode: Effective Charge Separation and Enhanced Photoelectrochemical Properties. J. Colloid Interface Sci..

[24] Liu Y., Ye Z., Li D., Wang M., Zhang Y., Huang W. (2019). Tuning CuOx -TiO_2_ Interaction and Photocatalytic Hydrogen Production of CuOx/TiO_2_ Photocatalysts via TiO_2_ Morphology Engineering. Appl. Surf. Sci..

[25] Asen P., Shahrokhian S. (2017). A High Performance Supercapacitor Based on Graphene/Polypyrrole/Cu_2_O-Cu(OH)_2_ Ternary Nanocomposite Coated on Nickel Foam. J. Phys. Chem. C.

[26] Djellabi R., Yang B., Adeel Sharif H.M., Zhang J., Ali J., Zhao X. (2019). Sustainable and Easy Recoverable Magnetic TiO_2_-Lignocellulosic Biomass@Fe_3_O_4_ for Solar Photocatalytic Water Remediation. J. Clean. Prod..

[27] Reddy N.R., Kumar A.S., Reddy P.M., Merum D., Kakarla R.R., Jung J.H., Joo S.W., Aminabhavi T.M. (2023). Sharp-Edged Pencil Type ZnO Flowers and BiOI Flakes Combined with Carbon Nanofibers as Heterostructured Hybrid Photocatalysts for the Removal of Hazardous Pollutants from Contaminated Water. J. Environ. Manag..

[28] Pradhan A.C., Uyar T. (2017). Morphological Control of Mesoporosity and Nanoparticles within Co_3_O_4_-CuO Electrospun Nanofibers: Quantum Confinement and Visible Light Photocatalysis Performance. ACS Appl. Mater. Interfaces.

[29] Praveen Kumar D., Lakshmana Reddy N., Srinivas B., Durgakumari V., Roddatis V., Bondarchuk O., Karthik M., Ikuma Y., Shankar M.V. (2016). Stable and Active CuxO/TiO_2_ nanostructured Catalyst for Proficient Hydrogen Production under Solar Light Irradiation. Sol. Energy Mater. Sol. Cells.

[30] Liu Q., Cao J., Ji Y., Liu Y., Liu C., Che G., Wang D., Cao J., Li W., Liu X. (2021). The Direct Z-Scheme Cd_x_Zn_1-X_S Nanorods-Fe_2_O_3_ Quantum Dots Heterojunction/Reduced Graphene Oxide Nanocomposites for Photocatalytic Degradation and Photocatalytic Hydrogen Evolution. Appl. Surf. Sci..

[31] Habibi-Yangjeh A., Mousavi M., Nakata K. (2019). Boosting Visible-Light Photocatalytic Performance of g-C_3_N_4_/Fe_3_O_4_ Anchored with CoMoO_4_ Nanoparticles: Novel Magnetically Recoverable Photocatalysts. J. Photochem. Photobiol. A Chem..

[32] Mousavi M., Habibi-Yangjeh A. (2018). Magnetically Recoverable Highly Efficient Visible-Light-Active g-C_3_N_4_/Fe_3_O_4_/Ag_2_WO_4_/AgBr Nanocomposites for Photocatalytic Degradations of Environmental Pollutants. Adv. Powder Technol..

[33] Reddy N.R., Bhargav U., Kumari M.M., Cheralathan K.K., Shankar M.V., Reddy K.R., Saleh T.A., Aminabhavi T.M. (2020). Highly Efficient Solar Light-Driven Photocatalytic Hydrogen Production over Cu/FCNTs-Titania Quantum Dots-Based Heterostructures. J. Environ. Manage..

[34] Reddy N.R., Kumar A.S., Reddy P.M., Reddy R., Woo S., Aminabhavi T.M. (2023). Novel Rhombus Co_3_O_4_ -Nanocapsule CuO Heterohybrids for Efficient Photocatalytic Water Splitting and Electrochemical Energy Storage Applications. J. Environ. Manage..

[35] Fang Y., Wang Y., Wang F., Shu C., Zhu J., Wu W. (2018). Fe-Mn Bimetallic Oxides-Catalyzed Oxygen Reduction Reaction in Alkaline Direct Methanol Fuel Cells. RSC Adv..

[36] May Y.A., Wei S., Yu W.Z., Wang W.W., Jia C.J. (2020). Highly Efficient CuO/α-MnO_2_ Catalyst for Low-Temperature CO Oxidation. Langmuir.

[37] Wang Y., Zhou M., He Y., Zhou Z., Sun Z. (2020). In Situ Loading CuO Quantum Dots on TiO_2_ Nanosheets as Cocatalyst for Improved Photocatalytic Water Splitting. J. Alloys Compd..

[38] Nallapureddy R.R., Pallavolu M.R., Nallapureddy J., Yedluri A.K., Joo S.W. (2023). Z-Scheme Photocatalysis and Photoelectrochemical Platform with a Co_3_O_4-_CuO Heterogeneous Catalyst for the Removal of Water Pollutants and Generation of Energy. J. Clean. Prod..

[39] Suresh R., Giribabu K., Manigandan R., Stephen A., Narayanan V. (2014). Fabrication of Ni-Fe_2_O_3_ Magnetic Nanorods and Application to the Detection of Uric Acid. RSC Adv..

[40] Zou D., Yi Y., Song Y., Guan D., Xu M., Ran R., Wang W., Zhou W., Shao Z. (2022). The BaCe_0.16_Y_0.04_Fe_0.8_O_3-d_ Nanocomposite: A New High-Performance Cobalt-Free Triple-Conducting Cathode for Protonic Ceramic Fuel Cells Operating at Reduced Temperatures. J. Mater. Chem. A.

[41] Li J., Li F., Qian M., Han M., Liu H., Zhang D., Ma J., Zhao C. (2017). Characteristics and Regulatory Pathway of the PrupeSEP1 SEPALLATA Gene during Ripening and Softening in Peach Fruits. Plant Sci..

[42] Lyu J., Ge M., Hu Z., Guo C. (2020). One-Pot Synthesis of Magnetic CuO/Fe_2_O_3_/CuFe_2_O_4_ Nanocomposite to Activate Persulfate for Levofloxacin Removal: Investigation of Efficiency, Mechanism and Degradation Route. Chem. Eng. J..

[43] Tan L., Xu J., Zhang X., Hang Z., Jia Y., Wang S. (2015). Synthesis of g-C_3_N_4_/CeO_2_ Nanocomposites with Improved Catalytic Activity on the Thermal Decomposition of Ammonium Perchlorate. Appl. Surf. Sci..

[44] Huang Z., Sun Q., Lv K., Zhang Z., Li M., Li B. (2015). Effect of Contact Interface between TiO_2_ and g-C_3_N_4_ on the Photoreactivity of g-C_3_N_4_/TiO_2_ Photocatalyst: (001) vs (101) Facets of TiO_2_. Appl. Catal. B Environ..

[45] Cao S.W., Yuan Y.P., Barber J., Loo S.C.J., Xue C. (2014). Noble-Metal-Free g-C_3_N_4_ /Ni(DmgH)_2_ Composite for Efficientphotocatalytic Hydrogen Evolution under Visible Light Irradiation. Appl. Surf. Sci..

[46] Kakinuma K., Suda K., Kobayashi R., Tano T., Arata C., Amemiya I., Watanabe S., Matsumoto M., Imai H., Iiyama A. (2019). Electronic States and Transport Phenomena of Pt Nanoparticle Catalysts Supported on Nb-Doped SnO_2_ for Polymer Electrolyte Fuel Cells. ACS Appl. Mater. Interfaces.

[47] Arunachalam M., Lee D.G., Das P.K., Subhash K.R., Ahn K.S., Kang S.H. (2022). Surface Engineering of Ba-Doped TiO_2_ Nanorods by Bi_2_O_3_ Passivation and (NiFe)OOH Co-Catalyst Layers for Efficient and Stable Solar Water Oxidation. Int. J. Hydrogen Energy.

[48] Chen Y.C., Yeh H.Y., Popescu R., Gerthsen D., Hsu Y.K. (2022). Solution–Processed Cu_2_O/ZnO/TiO_2_/Pt Nanowire Photocathode for Efficient Photoelectrochemical Water Splitting. J. Alloys Compd..

[49] Zheng Y., Ruan Q., Ren J.X., Guo X., Zhou Y., Zhou B., Xu Q., Fu Q., Wang S., Huang Y. (2023). Plasma- Assisted Liquid-Based Growth of g-C_3_N_4_/Mn_2_O_3_ p-n Heterojunction with Tunable Valence Band for Photoelectrochemical Application. Appl. Catal. B Environ..

[50] Zhang S., Yan J., Yang S., Xu Y., Cai X., Li X., Zhang X., Peng F., Fang Y. (2017). Electrodeposition of Cu_2_O/g-C_3_N_4_ Heterojunction Film on an FTO Substrate for Enhancing Visible Light Photoelectrochemical Water Splitting. Cuihua Xuebao/Chinese J. Catal..

[51] Moakhar R.S., Soleimani F., Sadrnezhaad S.K., Masudy-Panah S., Katal R., Seza A., Ghane N., Ramakrishna S. (2020). One-Pot Microwave Synthesis of Hierarchical C-Doped CuO Dandelions/g-C_3_N_4_ Nanocomposite with Enhanced Photostability for Photoelectrochemical Water Splitting. Appl. Surf. Sci..

[52] Parvari R., Ghorbani-Shahna F., Bahrami A., Azizian S., Assari M.J., Farhadian M. (2020). A Novel Core-Shell Structured α-Fe_2_O_3_/Cu/g-C_3_N_4_ Nanocomposite for Continuous Photocatalytic Removal of Air Ethylbenzene under Visible Light Irradiation. J. Photochem. Photobiol. A Chem..

[53] Reddy I.N., Sreedhar A., Shim J., Gwag J.S. (2019). Multifunctional Monoclinic VO_2_ Nanorod Thin Films for Enhanced Energy Applications: Photoelectrochemical Water Splitting and Supercapacitor. J. Electroanal. Chem..

[54] Chen M., Chang X., Li C., Wang H., Jia L. (2023). Ni-Doped BiVO_4_ Photoanode for Efficient Photoelectrochemical Water Splitting. J. Colloid Interface Sci..

[55] Wang J., Chen R., Xiang L., Komarneni S. (2018). Synthesis, Properties and Applications of ZnO Nanomaterials with Oxygen Vacancies: A Review. Ceram. Int..

[56] Li S., Wang L., Li Y.D., Zhang L., Wang A., Xiao N., Gao Y., Li N., Song W., Ge L. (2019). Novel Photocatalyst Incorporating Ni-Co Layered Double Hydroxides with P-Doped CdS for Enhancing Photocatalytic Activity towards Hydrogen Evolution. Appl. Catal. B Environ..

[57] Hao C., Wang W., Zhang R., Zou B., Shi H. (2018). Enhanced Photoelectrochemical Water Splitting with TiO_2_@Ag_2_O Nanowire Arrays via P-n Heterojunction Formation. Sol. Energy Mater. Sol. Cells.

[58] Bai S., Yang X., Liu C., Xiang X., Luo R., He J., Chen A. (2018). An Integrating Photoanode of WO_3_/Fe_2_O_3_ Heterojunction Decorated with NiFe-LDH to Improve PEC Water Splitting Efficiency. ACS Sustain. Chem. Eng..

[59] Chen L., Zuo X., Yang S., Cai T., Ding D. (2019). Rational Design and Synthesis of Hollow Co_3_O_4_@Fe_2_O_3_ Core-Shell Nanostructure for the Catalytic Degradation of Norfloxacin by Coupling with Peroxymonosulfate. Chem. Eng. J..

[60] Sitara E., Nasir H., Mumtaz A., Ehsan M.F., Sohail M., Iram S., Bukhari S.A.B., Ullah S., Akhtar T., Iqbal A. (2021). Enhanced Photoelectrochemical Water Splitting Using Zinc Selenide/Graphitic Carbon Nitride Type-II Heterojunction Interface. Int. J. Hydrogen Energy.

[61] Mustafa E., Dawi E.A., Ibupoto Z.H., Ibrahim A.M.M., Elsukova A., Liu X., Tahira A., Adam R.E., Willander M., Nur O. (2023). Efficient CuO/Ag_2_WO_4_ Photoelectrodes for Photoelectrochemical Water Splitting Using Solar Visible Radiation. RSC Adv..

[62] Yun X., Lei Y., Wang Z., Bo X., Ma Y. (2025). Highly Enhanced Photoelectrocatalytic Activity of NiFe/Ni/BiVO4 Photoanode by a Facile Photoelectron-Activation Process in Neutral Solution. J. Photochem. Photobiol. A Chem..

[63] Ding Y., Huang L., Barakat T., Su B.L. (2021). A Novel 3DOM TiO_2_ Based Multifunctional Photocatalytic and Catalytic Platform for Energy Regeneration and Pollutants Degradation. Adv. Mater. Interfaces.

[64] John S., Roy S.C. (2020). CuO/Cu_2_O nanoflake/nanowire heterostructure photocathode with enhanced surface area for photoelectrochemical solar energy conversion. Appl. Surf. Sci..

[65] Tian J., Li H., Xing Z., Wang L., Luo Y., Asiri A.M., Al-Youbi A.O., Sun X. (2012). One-pot green hydrothermal synthesis of CuO–Cu_2_O–Cu nanorod-decorated reduced graphene oxide composites and their application in photocurrent generation. Catal. Sci. Technol..

[66] Mahmood A., Tezcan F., Kardaş G. (2017). Molybdenum disulfide as the interfacial layer in the CuO–TiO_2_ photocathode for photoelectrochemical cells. J. Mater. Sci. Mater. Electron..

[67] Borkar R., Dahake R., Rayalu S., Bansiwal A. (2017). Copper Oxide Nanograss for Efficient and Stable Photoelectrochemical Hydrogen Production by Water Splitting. J. Electron. Mater..

[68] Arzaee N.A., Noh M.F.M., Ita N.S.H.M., Mohamed N.A., Nasir S.N.F.M., Mumthas I.N.N., Ismail A.F., Teridi M.A.M. (2020). Nanostructure-assisted charge transfer in α-Fe_2_O_3_/g-C3N4 heterojunctions for efficient and highly stable photoelectrochemical water splitting. Dalt. Trans..

[69] Liu Y., Su F.-Y., Yu Y.-X., Zhang W.-D. (2016). Nano g-C_3_N_4_ modified Ti-Fe_2_O_3_ vertically arrays for efficient photoelectrochemical generation of hydrogen under visible light. Int. J. Hydrogen Energy.

[70] Lei N., Li J., Song Q., Liang Z. (2019). Construction of g-C_3_N_4_/BCN two-dimensional heterojunction photoanode for enhanced photoelectrochemical water splitting. Int. J. Hydrogen Energy.

[71] Ragupathi V., Raja M.A., Panigrahi P., Subramaniam N.G. (2020). CuO/g-C_3_N_4_ nanocomposite as promising photocatalyst for photoelectrochemical water splitting. Optik.

[72] Li X., Wang Z., Zhang Z., Chen L., Cheng J., Ni W., Wang B., Xie E. (2015). Light Illuminated α-Fe_2_O_3_/Pt Nanoparticles as Water Activation Agent for photoelectrochemical water splitting. Sci. Rep..

