# Supplementary material for: Photosensitizer and Charge Separator Roles of g-C₃N₄ Integrated into the CuO-Fe₂O₃ p-n Heterojunction Interface for Elevating PEC Water Splitting Potential
Source: Nanomaterials (Basel). 2025 Apr 4;15(7):551. doi: 10.3390/nano15070551 (PMC11990803; doi:10.3390/nano15070551)
Supplement: Supplementary file 1 [file nanomaterials-15-00551-s001.zip › nanomaterials-3537165-supplementary.pdf]

Supplementary Materials

Photosensitizer and Charge Separator Roles of g-C_3N_4 Integrated into the CuO-Fe_2O_3 p-n Heterojunction Interface for Elevating PEC Water Splitting Potential

Ramesh Reddy Nallapureddy [1], Sai Kumar Arla [2], Andrés Ibáñez [3], Durga Prasad Pabba [4,*], Jae Hak Jung [1,*] and Sang Woo Joo [5,*]

[1] School of Chemical Engineering, Yeungnam University, Gyeongsan 38541, Republic of Korea; rameshsun999@gmail.com
[2] Department of Physics, Yeungnam University, Gyeongsan 38541, Republic of Korea; saiarla853@gmail.com
[3] Departamento de Física, Facultad de Ciencias Físicas y Matemáticas, Universidad de Chile, Casilla 653, Santiago 8370451, Chile; aibanez@dfi.uchile.cl
[4] Departamento de Electricidad, Facultad de Ingeniería, Universidad Tecnológica Metropolitana (UTEM), Santiago 7800002, Chile
[5] School of Mechanical Engineering, Yeungnam University, Gyeongsan 38541, Republic of Korea
* Correspondence: dpabba@utem.cl (D.P.P.), jhjung@ynu.ac.kr (J.H.J.); swjoo@yu.ac.kr (S.W.J.)

Academic Editor: Vincenzo Vaiano

Received: 4 March 2025
Revised: 28 March 2025
Accepted: 2 April 2025
Published: 4 April 2025

Citation: Reddy, N.R.; Arla, S.K.; Ibáñez, A.; Pabba, D.P.; Jung, J.H.; Joo, S.W. Photosensitizer and Charge Separator Roles of g-C_3N_4 Integrated into the CuO-Fe_2O_3 p-n Heterojunction Interface for Elevating Photo Electrochemical Water-Splitting Potential. *Nanomaterials* **2025**, *15*, 551. https://doi.org/10.3390/nano15070551

Copyright: © 2025 by the authors. Submitted for possible open access publication under the terms and conditions of the Creative Commons Attribution (CC BY) license (https://creativecommons.org/licenses/by/4.0/).

2.1. Material and Chemicals

Copper(II) acetate hydrate ($C_6H_6CuO_4$) was used as a copper precursor and purchased from Sigma-Aldrich (purity: 98.0%). Iron(III) chloride hexahydrate ($FeCl_3{\cdot}6H_2O$) served as an iron precursor and was obtained from Sigma-Aldrich (purity: ≥ 98.5%). ($C_6H_{12}N_4$) was utilized as a structural agent and purchased from Dae Jung (purity: > 98%). Ethanol was employed for material washing and was purchased from Duksan reagents (purity: 99.9%).

2.3. Characterization details

The crystallinity of the synthesized catalyst was investigated using high-intensity monochromatic Cu K radiation and X-ray diffraction (MPD for bulk, 3 kW, Panalytical) with a resolution of 0.15406 nm. Raman spectra in the range of 100–2500 cm^{-1} were obtained using a Horiba Jobin Yvon HR 800 UV confocal micro-Raman spectrometer equipped with a Nd: YAG laser source operating in backscattering mode at a wavelength of 532 nm. Morphological characteristics, such as size, shape, and distribution, were examined using scanning electron microscopy (SEM; Hitachi S-4800) and transmission electron microscopy (TEM; Tecnai G2 F20 STwin). The elemental composition and oxidation states were investigated through X-ray photoelectron spectroscopy (XPS; Thermo Scientific) employing monochromatic Al Ka radiation. Additionally, elemental mapping, facilitated by the FE-SEM instrument, was utilized to assess the dopant distribution in the synthesized nanostructures. UV–vis DRS analysis of the prepared materials was performed using a Cari 5000 UV–Vis– dual-beam spectrometer with the wavelength varying from 300 to 1000 nm. FL spectra were measured using a fluorophotometer (RF-6000, Shimadzu) with a quartz cuvette.

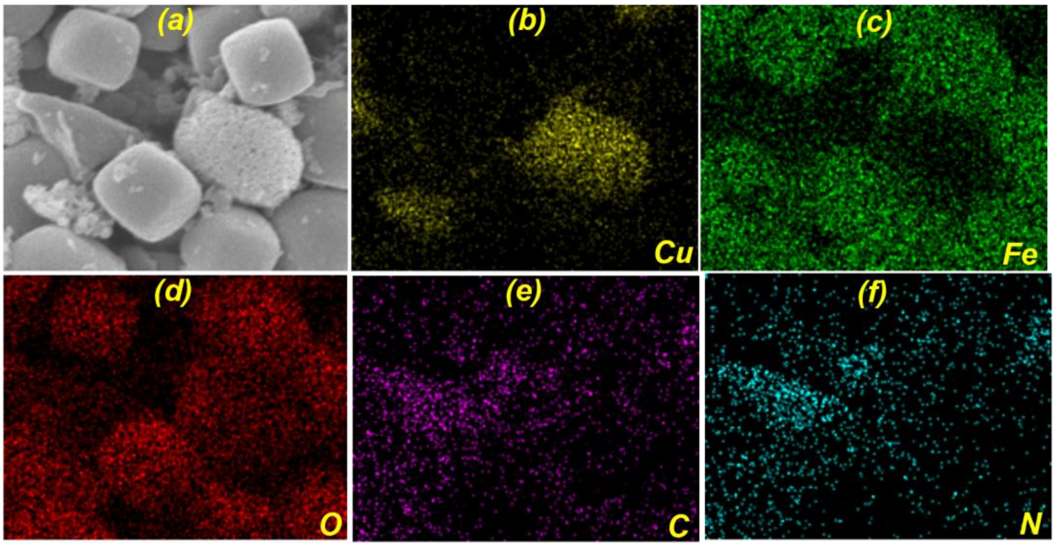

Figure S1. EDX elemental mapping of the CuO–Fe_2O_3@g-C_3N_4 nanocomposite. (**a**) Electron micrograph; (**b**–**f**) Distribution of Cu, Fe, O, C, and N elements.

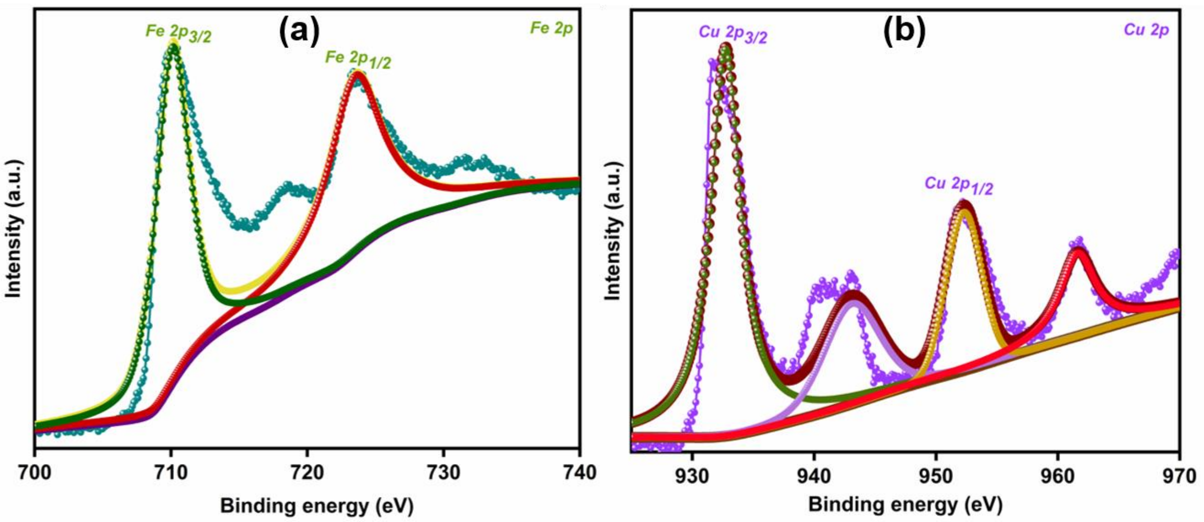

Figure S2. Deconvoluted XPS spectra (**a**) Fe 2p of pristine Fe_2O_3 and (**b**) Cu 2p of pristine CuO.

Table S1. Comparison of CuO, Fe_2O_3, and g-C_3N_4-based photoelectrodes with the present work.

Photoelectrode	Morphology	Synthesis process	Photocurrent density (vs Ag/AgCl)	Ref
CuO/Cu_2O	Nanoflake/nanowire Core-shell heterostructure	electrochemical anodization + annealing	1.9 mA cm^{-2} at 0.3 V	[1]
CuO-Cu_2O-Cu@rGO	Nanorods	Hydrothermal method	0.23 μA at 0 V	[2]
CuO/MoS_2/TiO_2		Galavanostatic deposition	-0373 mA cm^{-2} at -0.55 V	[3]
CuO/Cu_2O	Grass appendage	Electrodeposition	1.44 mA cm^{-2} at -0.7 V	[4]
◎-Fe_2O_3/g-C_3N_4	Film	Aerosol-assisted chemical vapour deposition	1.17 mA cm^{-2} at 1.2 V	[5]
g-C_3N_4/Ti-Fe_2O_3	Nanosheet arrays	Electrodeposition	2.5 mA cm^{-2} at 0.23 V	[6]
CuO-Ag_2WO_4	Nanoparticles	Spin coating and SILAR	-1.1 mA cm^{-2} at -0.55 V	[7]
g-C_3N_4/BCN	**2D/2D** nanosheet heterostructure	Thermal polymerization	1.15 mA cm^{-2} at 1.6 V	[8]
CuO/g-C_3N_4	**CuO nanorods**	Co-precipitation	0.68 mA cm^{-2} at 1.6 V	[9]

◎-Fe_2O_3/pt	**Nanoparticles**	Wet chemical	0.345 mA cm^{-2} at 1.6 V	[10]
◎-Fe_2O_3/CuO	**Porous CuO**	Dip-coating	0.53 mA cm^{-2} at 1.6 V	[11]
**CuO-Fe_2O_3@g-C_3N_4**	**Nanospheres and micro-cubes**	**Hydrothermal method**	**1.33 mA cm^{-2} at 1.6 V**	**This work**

References

1. John, S.; Roy, S.C. CuO/Cu_2O nanoflake/nanowire heterostructure photocathode with enhanced surface area for photoelectrochemical solar energy conversion. *Appl. Surf. Sci.* **2020**, *509*, 144703. https://doi.org/10.1016/j.apsusc.2019.144703.
2. Tian, J.; Li, H.; Xing, Z.; Wang, L.; Luo, Y.; Asiri, A.M.; Al-Youbi, A.O.; Sun, X. One-pot green hydrothermal synthesis of CuO–Cu_2O–Cu nanorod-decorated reduced graphene oxide composites and their application in photocurrent generation. *Catal. Sci. Technol.* **2012**, *2*, 2227–2230, https://doi.org/10.1039/c2cy20406a.
3. Mahmood, A.; Tezcan, F.; Kardaş, G. Molybdenum disulfide as the interfacial layer in the CuO–TiO_2 photocathode for photoelectrochemical cells. *J. Mater. Sci. Mater. Electron.* **2017**, *28*, 12937–12943, https://doi.org/10.1007/s10854-017-7124-y.
4. Borkar, R.; Dahake, R.; Rayalu, S.; Bansiwal, A. Copper Oxide Nanograss for Efficient and Stable Photoelectrochemical Hydrogen Production by Water Splitting. *J. Electron. Mater.* **2017**, *47*, 1824–1831, https://doi.org/10.1007/s11664-017-5966-y.
5. Arzaee, N.A.; Noh, M.F.M.; Ita, N.S.H.M.; Mohamed, N.A.; Nasir, S.N.F.M.; Mumthas, I.N.N.; Ismail, A.F.; Teridi, M.A.M. Nanostructure-assisted charge transfer in α-Fe_2O_3/g-C3N4 heterojunctions for efficient and highly stable photoelectrochemical water splitting. *Dalt. Trans.* 2020, 49, 11317–11328.
6. Liu, Y.; Su, F.-Y.; Yu, Y.-X.; Zhang, W.-D. Nano g-C_3N_4 modified Ti-Fe_2O_3 vertically arrays for efficient photoelectrochemical generation of hydrogen under visible light. *Int. J. Hydrogen Energy* **2016**, *41*, 7270–7279, https://doi.org/10.1016/j.ijhydene.2016.03.113.
7. Mustafa, E.; Dawi, E.A.; Ibupoto, Z.H.; Ibrahim, A.M.M.; Elsukova, A.; Liu, X.; Tahira, A.; Adam, R.E.; Willander, M.; Nur, O. Efficient CuO/Ag_2WO_4 Photoelectrodes for Photoelectrochemical Water Splitting Using Solar Visible Radiation. *RSC Adv.* **2023**, *13*, 11297–11310.
8. Lei, N.; Li, J.; Song, Q.; Liang, Z. Construction of g-C_3N_4/BCN two-dimensional heterojunction photoanode for enhanced photoelectrochemical water splitting. *Int. J. Hydrogen Energy* **2019**, *44*, 10498–10507, https://doi.org/10.1016/j.ijhydene.2019.02.160.
9. Ragupathi, V.; Raja, M.A.; Panigrahi, P.; Subramaniam, N.G. CuO/g-C_3N_4 nanocomposite as promising photocatalyst for photoelectrochemical water splitting. *Optik* **2020**, *208*, https://doi.org/10.1016/j.ijleo.2020.164569.
10. Li, X.; Wang, Z.; Zhang, Z.; Chen, L.; Cheng, J.; Ni, W.; Wang, B.; Xie, E. Light Illuminated α-Fe_2O_3/Pt Nanoparticles as Water Activation Agent for photoelectrochemical water splitting. *Sci. Rep.* **2015**, *5*, 9130.
11. Kyesmen, P.I.; Nombona, N.; Diale, M. Heterojunction of Nanostructured α-Fe_2O_3/CuO for Enhancement of Photoelectrochemical Water Splitting. *J. Alloys Compd.* **2021**, *863*, 158724.

Disclaimer/Publisher's Note: The statements, opinions and data contained in all publications are solely those of the individual author(s) and contributor(s) and not of MDPI and/or the editor(s). MDPI and/or the editor(s) disclaim responsibility for any injury to people or property resulting from any ideas, methods, instructions or products referred to in the content.
